# Impact of diabetes-related gene polymorphisms on the clinical characteristics of type 2 diabetes Chinese Han population

**DOI:** 10.18632/oncotarget.13399

**Published:** 2016-11-16

**Authors:** Jing Li, Jiache Wei, Pengcheng Xu, Mengdan Yan, Jingjie Li, Zhengshuai Chen, Tianbo Jin

**Affiliations:** ^1^ Key Laboratory of Resource Biology and Biotechnology in Western China (Northwest University), Ministry of Education, School of Life Sciences, Northwest University, Xi’an, Shaanxi 710069, China; ^2^ Department of Endocrinology, Xi’an NO.1 Hospital, Xi’an 710002, China; ^3^ Inner Mongolia Medical University, Inner Mongolia, Hohhot 010010, China; ^4^ Xi’an Tiangen Precision Medical Institute, Xi’an, Shaanxi, 710075, China

**Keywords:** type2 diabetes, single nucleotide polymorphism, case-control, genetic

## Abstract

We investigated the correlation between type 2 diabetes (T2D)-related genes and the clinical characteristics of T2D in the Chinese Han population. Our study included 319 patients and 387 controls. Age, gender, clinical features, medications intake and biochemical blood profiles were analyzed. Genotyping was performed on a total of 18 single nucleotide polymorphisms previously reported to be associated with T2D. Our analyses revealed that the CT genotype of *ARHGAP22* rs4838605 is associated with T2D risk. Upon analyzing the subjects’ clinical characteristics, we found that for rs2811893, the TT genotype correlated with high creatinine levels, while the AA genotype of rs17045754 and the TT genotype of rs4838605 correlated with elevated triglyceride levels. In addition, the AA genotype of rs17376456 and the TT genotype of rs6214 (*p* = 0.006) correlated with elevated hemoglobin A1c levels. Lastly, those carrying the TT genotype of rs7772697 and the CA genotype of rs3918227 exhibited higher mean body mass index and Cystatin C than controls. Our results showing that the *ARHGAP22* gene is associated with an increased risk of T2D, and that seven SNPs in *MYSM1*, *PLXDC2*, *ARHGAP22* and *HS6ST3* promote T2D progression and could help predict the clinical course of T2D in patients at risk.

## INTRODUCTION

Hallmarks of type 2 diabetes (T2D) include insulin resistance and reduced insulin secretion [1–4]. Alteration of glucose metabolism is the primary cause of T2D [5, 6]. In early stages, T2D is often asymptomatic and can remain undetected for several years [7]. According to the International Diabetes Federation (IDF), as of April 2016 415 million people worldwide had diabetes, a number that will rise to 642 million (http://www.diabetesatlas.org/) by 2040.

Previous studies have shown that T2D risk is largely determined by lifestyle habits including active smoking [8], quality of sleep [9], weight loss [10], fruit and vegetable intake [11], and coffee consumption [12]. At the same time, hundreds of studies reported associations between single nucleotide polymorphisms (SNPs) and T2D risk (http://diabetes.diabetesjournals.org). Genetic susceptibility to T2D risk is multifactorial. In the past few years, many additional T2D risk loci have been identified including *TCF7L2, FTO, HHEX, SLC30A8, HMG20A, IGF2BP2, MYSM1*, *TNFSF4*, *MYT1L*, *KIAA0825*, *VEGFA*, *PLXDC2*, *CREB5*, *NOS3*, *ARHGAP22*, *IGF1* [13–21]. However, for many of the reports the association between SNPs and T2D risk might have been false-positives or of minor impact in different populations. Indeed, single population studies might not fully explain associations between SNPs of candidate genes and T2D risk.

Early T2D diagnosis can reduce long-term complications, such as diabetic retinopathy, kidney failure, cardiovascular disease and limb amputation [22–25]. Unfortunately, screening tests to measure T2D risk are not consistent. The most widely used screening tests include the fasting plasma glucose (FPG) test, glycosylated hemoglobin A1c (HbA1c) test, and the oral glucose tolerance test (OGTT) [26]. We propose that screening methods can be improved by combining analyses of genetic polymorphisms with a lifestyle patterns to predict T2D. In this study, our aim was to investigate the relationship between newly discovered genes associated with T2D susceptibility and the clinical characteristic of T2D in the Chinese Han population.

## RESULTS

### Demographic and clinical profile of cases and controls

The demographic characteristics of the participants are presented in Table 1. The cases and controls were matched by sex, but there was a difference in age between cases and controls (*p* = 0.021), averaging 58.89 ± 12.01 and 57.05 ± 8.25 years, respectively.

**Table 1:** Basic characteristics of diabetes patients and controls

Characteristics		Case	Control	*p-*value
**Demographic**				
Number		319	387	
Gender				0.258
female		143	190	
male		176	197	
Age(year)	( mean ± SD)	58.89±12.01	57.05±8.25	0.021*
**Clinical**				
BMI, kg/m2	(mean ± SD)	24.92±3.44		
Duration of diabetes(year)	(mean ± SD)	9.37±7.76		
Fasting glucose(mmol/L)	(mean ± SD)	9.82±4.53		
Complication	Negative	84		
	Positive	235		
Smoker	Negative	221		
	Positive	98		
Drinker	Negative	266		
	Positive	53		
**Biochemical**				
HbA1c(mmol/L)	(mean ± SD)	9.25±2.33		
Total cholesterol(mmol/L)	(mean ± SD)	4.64±1.27		
Triglycerides(mmol/L)	(mean ± SD)	2.53±2.21		
LDL cholesterol(mmol/L)	(mean ± SD)	2.78±0.91		
HDL cholesterol(mmol/L)	(mean ± SD)	1.20±0.53		
Urea(mmol/L)	(mean ± SD)	6.27±2.38		
Cr(mmol/L)	(mean ± SD)	65.47±28.32		
Cys-c(mmol/L)	(mean ± SD)	0.87±0.42		
GFR(mmol/L)	(mean ± SD)	123.70±34.18		
CP(mmol/L)	(mean ± SD)	1.40±1.48		
INS(mmol/L)	(mean ± SD)	18.73±17.48		
UCRP(mmol/L)	(mean ± SD)	0.52±1.14		
**Medications**				
Hypoglycemic drugs	Negative	194		
	Positive	125		
Patients on insulin therapy	Negative	156		
	Positive	163		

*p* < 0.05 indicates statistical significance;

Continuous variables are expressed as mean ± SD;

Comparisons between groups were performed using ANOVA for continuous variables and x^2^test for categorical variables.

### Characteristic of SNPs in the study population

The basic information of candidate SNPs in our study including gene name, chromosome, minor allele frequency (MAF), and Hardy–Weinberg Equilibrium (HWE) test, appears in Table 2. SNPs at MAF < 0.05 were removed from association analyses. The HWE agreed with *p* > 0.05. However, we identified no significant association between SNPs and T2D risk under the allelic model (Table 2).

**Table 2:** Basic information on candidate SNPs

SNP ID	Genes	Chromosome	Alleles A/B	MAF-case	MAF-control	HWE test *p*-value	ORs	95%CI		p*
rs3007729	Unknown	1	T/C	T	C	1.000	1.23	1.00	1.53	0.052
rs2811893	MYSM1	1	C/T	C	T	0.230	0.95	0.76	1.18	0.633
rs1342038	TNFSF4	1	G/A	G	A	0.599	0.88	0.71	1.08	0.224
rs10927101	Unknown	1	C/A	C	A	0.909	1.01	0.81	1.26	0.925
rs10199521	MYT1L	2	T/C	T	C	0.897	1.18	0.94	1.49	0.157
rs13064954	Unknown	3	A/G	A	G	0.288	0.95	0.67	1.33	0.758
rs17376456	KIAA0825	5	G/A	G	A	0.713	0.79	0.52	1.20	0.267
rs3025040	VEGFA	6	T/C	T	C	0.731	0.86	0.65	1.14	0.302
rs7772697	Unknown	6	C/T	C	T	0.346	1.06	0.80	1.41	0.671
rs11765845	CREB5	7	A/G	A	G	1.000	0.98	0.77	1.24	0.872
rs1799983	NOS3	7	T/G	T	G	0.083	1.13	0.81	1.59	0.471
rs3918227	NOS3	7	A/C	A	C	0.222	1.18	0.79	1.77	0.426
rs1571942	PLXDC2	10	G/A	G	A	1.000	1.05	0.74	1.50	0.779
rs12219125	Unknown	10	T/G	T	G	1.000	1.07	0.75	1.52	0.716
rs4838605	ARHGAP22	10	C/T	C	T	0.396	1.24	0.89	1.74	0.207
rs6219	IGF1	12	T/C	T	C	0.601	1.13	0.86	1.48	0.374
rs6214	IGF1	12	T/C	T	C	0.839	0.90	0.73	1.11	0.332
rs10403021	Unknown	19	T/C	T	C	0.706	1.14	0.91	1.44	0.264

MAF = minor allele frequency; HWE = Hardy–Weinberg Equilibrium;

OR= odds ratio; 95% CI =95 % confidence interval.

### Relationships between gene polymorphisms and clinical features

Genes polymorphisms were also analyzed to establish their associations with clinical features, including gender, age, BMI, duration of diabetes (year), fasting glucose, complications, biochemical criterion, drinking and smoking status, and hypoglycemic drug consumption. We investigated the relationship between candidate gene polymorphisms and the various clinical features listed in patients with T2D. Results for *MYSM1*, *KIAA0825*, *NOS3*, *ARHGAP2*, and *IGF1* were listed in Table 3. For rs2811893, the TT genotype appeared to have high Cr levels in cases (mean ± SD = 71.18 ± 38.63, *p* = 0.006), compared with the TC+CC genotype. The AA genotype of rs17045754 and the TT genotype of rs4838605 exhibited higher levels of triglycerides (TG) (mean ± SD = 7.59 ± 5.17, *p* = 0.001 and mean ± SD = 2.66 ± 2.36, *p* = 0.041, respectively). Moreover, The AA genotype of rs17376456 and the TT genotype of rs6214 are associated with high levels of hemoglobin A1C (HbA1C) (mean ± SD = 9.35 ± 2.38, *p* = 0.039 and mean ± SD =10.10 ± 3.34, *p* = 0.006, respectively). We also found that the TT genotype of rs7772697 and the CA genotype of rs3918227 had a higher mean BMI (mean ± SD = 25.25 ± 3.48, *p* = 0.028) and Cystatin C (Cys-c) (mean ± SD = 1.02 ± 0.91, *p* = 0.045), respectively (Table 3).

**Table 3:** The associations between genetic polymorphisms and clinical characteristics of T2D

SNP	genotype	n	HbA1c		TG		Cr		Cys-c		BMI	
			mean ± SD	p*	mean ± SD	p*	mean ± SD	p*	mean ± SD	p*	mean ± SD	p*
rs2811893(MYSM1)	TT	130	9.56±2.81	0.083	2.53±2.20	0.906	71.18±38.63	0.006*	0.87±0.26	0.975	24.79±3.59	0.495
	CT	148	9.15±1.98		2.51±2.19		61.95±18.39		0.87±0.56		24.90±3.36	
	CC	40	8.68±1.66		2.68±2.37		11.93±1.89		0.86±0.17		25.52±3.24	
rs13064954(Unknown)	AA	4	10.95±2.03	0.343	7.59±5.17	0.001*	66.47±21.70	0.584	0.85±0.20	0.615	25.27±3.94	0.555
	GG	257	9.22±2.40		2.53±2.20		64.67±27.78		0.85±0.23		24.82±3.52	
	AG	58	9.25±1.99		2.22±1.51		68.94±31.10		0.97±0.84		25.35±3.06	
rs17376456(KIAA0825)	AA	280	9.35±2.38	0.039*	2.56±2.91	0.6	65.56±29.67	0.88	0.87±0.44	0.526	24.92±3.49	0.884
	GA	39	8.53±1.78		2.36±1.49		64.83±15.70		0.83±0.20		25.00±3.44	
rs7772697(Unknown)	TT	223	9.18±2.40	0.455	2.61±2.38	0.584	65.88±29.90	0.916	0.88±0.48	0.779	25.25±3.48	0.028*
	CT	85	9.51±2.26		2.39±1.83		64.48±25.11		0.85±0.19		24.09±3.34	
	CC	11	8.85±1.23		2.08±0.90		63.29±18.46		0.83±0.30		24.69±2.28	
rs3918227(NOS3)	CA	49	8.96±1.74	0.338	2.00±1.07	0.117	63.85±18.03	0.664	1.02±0.91	0.045*	25.09±3.53	0.075
	CC	270	9.31±2.42		2.63±2.35		65.76±29.82		0.84±0.23		24.89±3.43	
rs4838605(ARHGAP22)	TT	243	9.34±2.43	0.216	2.66±2.36	0.041*	65.77±23.91	0.737	0.89±0.46	0.168	24.95±3.28	0.777
	TC	76	8.97±1.97		2.13±1.56		64.51±39.42		0.81±0.25		24.82±3.92	
rs6214(IGF1)	TT	72	10.10±3.34	0.006*	2.51±2.36	0.155	61.03±15.20	0.319	0.85±0.27	0.571	25.10±3.43	0.803
	CC	105	8.98±1.86		2.22±1.60		66.57±26.55		0.90±0.63		24.76±3.51	
	TC	142	9.03±1.91		2.78±2.48		66.90±34.05		0.85±0.26		24.96±3.41	

HbA1C = glycosylated hemoglobin A1c; TG = triglyceride; Cr = creatinine; Cys-c = cystatin C; BMI= body mass index;

OR = odds ratio; 95%CI = 95 % confidence interval;

*p ≤ 0.05 indicates statistical significance.

### Association of genotype and genetic models with T2D risk

The observed genotypes of the above seven SNPs for cases were compared to those of controls. The genotype frequencies of the seven gene polymorphisms *MYSM1* (rs2811893), *LEKR1-CCNL1* (rs13064954), *KIAA0825* (rs17376456), *UST-TAB2* (rs7772697), *NOS3* (rs3918227), *ARHGAP22* (rs4838605), and *IGF1* (rs6214) were shown in Table 4. The rs4838605 genotype frequency distribution in T2D patients was: TT, CT, CC. The CT genotype increased the risk of T2D compared with TT (CT vs TT: OR = 1.54, 95% CI = 1.06–2.24, *p* = 0.003). Furthermore, we assumed that the minor allele of each SNP was a risk allele compared to the wild type allele. Four genetic models including log-additive, over-dominant, dominant and recessive were applied to analyze the associations between the SNPs and T2D risk using a logistic regression test in Table 5. We found that the minor allele “C” of rs4838605 has an association with increased T2D risk under the overdominant model (OR = 1.57; 95% CI = 1.08–2.27; *p* = 0.018) (Table 5). In order to assess the associations between SNP haplotypes and T2D risk, a Wald test was performed using an unconditional multivariate regression analysis. However, no positive results were observed (data not shown).

**Table 4:** The polymorphisms of genotype model in the cases and controls and the associations with T2D risk (adjust by age and gender)

SNP	Genotype	Control	Case	OR (95% CI)	*p*-value
rs2811893	T/T	159 (41.1%)	130 (40.9%)	1	0.470
	C/T	169 (43.7%)	148 (46.5%)	1.08 (0.78-1.48)	
	C/C	59 (15.2%)	40 (12.6%)	0.81 (0.51-1.29)	
rs13064954	G/G	305 (78.8%)	257 (80.6%)	1	0.420
	G/A	80 (20.7%)	58 (18.2%)	0.87 (0.60-1.27)	
	A/A	2 (0.5%)	4 (1.2%)	2.46 (0.44-13.64)	
rs17376456	A/A	329 (85%)	280 (87.8%)	1	0.310
	G/A	57 (14.7%)	39 (12.2%)	0.79 (0.51-1.23)	
	G/G	1 (0.3%)	0 (0%)	0.00 (0.00-NA)	
rs7772697	T/T	270 (70%)	223 (69.9%)	1	0.360
	C/T	109 (28.2%)	85 (26.6%)	0.92 (0.66-1.29)	
	T/T	7 (1.8%)	11 (3.5%)	1.88 (0.71-4.97)	
rs3918227	C/C	339 (87.6%)	270 (84.6%)	1	0.046
	C/A	45 (11.6%)	49 (15.4%)	1.41 (0.91-2.20)	
	A/A	3 (0.8%)	0 (0%)	0.00 (0.00-NA)	
rs4838605	T/T	316 (81.7%)	243 (76.2%)	1	0.003*
	C/T	66 (17.1%)	76 (23.8%)	1.54 (1.06-2.24)	
	C/C	5 (1.3%)	0 (0%)	0.00 (0.00-NA)	
rs6214	C/C	108 (27.9%)	105 (32.9%)	1	0.310
	C/T	191 (49.4%)	142 (44.5%)	0.76 (0.54-1.08)	
	T/T	88 (22.7%)	72 (22.6%)	0.84 (0.56-1.28)	

CI = confidence interval; OR = odds ratio; SNP = single nucleotide polymorphism;

*p ≤ 0.05 indicates statistical significance.

**Table 5:** Single-SNP analysis and in different genetic models after adjusted by age & gender

SNP	Minor Allele	Dominant model				Recessive model				Over-dominant model				Log-additive model			
		OR	95% CI		*p*	OR	95% CI		*p*	OR	95% CI		*p*	OR	95% CI		*p*
rs2811893	C	1.01	0.74	1.36	0.970	0.78	0.5	1.2	0.250	1.14	0.84	1.53	0.400	0.94	0.76	1.17	0.580
rs13064954	A	0.91	0.63	1.32	0.610	2.53	0.46	14	0.270	0.86	0.59	1.26	0.440	0.96	0.68	1.36	0.800
rs17376456	G	0.78	0.5	1.21	0.260	NA	NA	NA	NA	0.8	0.51	1.24	0.310	0.77	0.5	1.18	0.230
rs7772697	C	0.98	0.7	1.35	0.880	1.93	0.73	5.06	0.180	0.9	0.64	1.26	0.530	1.04	0.78	1.39	0.780
rs3918227	A	1.32	0.85	2.03	0.210	NA	NA	NA	NA	1.43	0.92	2.22	0.110	1.21	0.8	1.83	0.370
rs4838605	C	1.43	0.99	2.06	0.056	NA	NA	NA	NA	1.57	1.08	2.27	0.018*	1.28	0.91	1.82	0.160
rs6214	T	0.79	0.57	1.09	0.150	1	0.7	1.42	0.980	0.82	0.61	1.1	0.190	0.91	0.74	1.11	0.350

OR = odds ratio; 95%CI = 95 % confidence interval;

*p ≤ 0.05 indicates statistical significance.

## DISCUSSION

There are several biochemical markers for T2D including Cr, TG, HbA1C and Cys-c levels. An interplay between genetic and environmental factors seems to determine T2D risk. In this study, we selected previously published genetic loci associated with diabetic retinopathy to investigate their relationship with T2D risk and clinical characteristics of T2D in the Chinese Han population. Our results suggest that *MYSM1*, *PLXDC2*, *ARHGAP22* and *HS6ST3* might promote T2D, with *ARHGAP22* showing the strongest association with T2D risk (*p* = 0.018). In addition, we found significant correlations between SNPs and clinical features including (1) rs2811893 (*MYSM1*) T to C change and Cr levels; (2) A allele in rs13064954 and C allele in rs4838605 (*ARHGAP22*) polymorphism and TG levels; (3) T allele in rs6214 (*IGF1*) and G allele in rs17376456 (*KIAA0825*) polymorphism and HbA1c levels; (4) C allele in rs7772697 (unknown) polymorphism and BMI; and (5) A allele in rs3918227 (*NOS3*) polymorphism and Cys-c levels.

We investigated the association between 18 SNPs and T2D risk. We found that the rs4838605 (*ARHGAP22*) gene is associated with increased T2D risk (*p* = 0.018), consistent with previous studies. McAuley *et al.* found that diabetic retinopathy patients with rs4838605 polymorphism have a 1.58- fold increase in T2D risk under the co-dominant model [27]. Yu-Chuen Huang *et al*. provided the first evidence that *ARHGAP22* polymorphisms were involved in endothelial cell angiogenesis [17]. *ARHGAP22* encodes for a GTPase protein in the Rho family, which is involved in the signal transduction pathway that regulates endothelial cell capillary tube formation during angiogenesis [28, 29]. Recently, ARHGAP22 levels have been proposed to determine tumor cell movement [30, 31] and the *ARHGAP22* gene has been implicated in a novel insulin-regulated pathway [32, 33]. In our study, *ARHGAP22* was the only gene associated with T2D risk, which suggests that *ARHGAP22* might function as an insulin regulator.

Individuals with high levels of Cr, TG, Cys-c or HbA1C are more susceptible to T2D. BMI is also strongly associated with T2D risk [34]. Here, the variant rs17376456 and rs6214 genotypes presented elevated HbA1c levels and rs2811893 correlated with high Cr levels. Furthermore, the variant genotypes of rs13064954 and rs4838605 exhibited elevated TG levels. For the rs3918227 polymorphism, Cys-c levels were associated with increased T2D risk and CA genotype. Lastly, the rs7772697 polymorphism correlated with BMI status. These results suggest that the aforementioned SNPs promoted the development and progression of T2D and may help to predict the clinical course of T2D.

Diabetic nephropathy, predominantly due to T2D, is the most frequent cause of renal failure in the United States and in Europe [35, 36]. Therefore, prevention or delay of diabetic renal disease could improve the life of patients at risk [37]. Glomerular filtration rate (GFR) is the best overall index of renal function. Cys-C has been proposed as a reliable serum marker for GFR [38–41]. In addition, Oddoze *et al.* found that serum Cr can estimate the GFR in diabetic patients [42]. In this study, we found that rs2811893 in the *MYSM1* gene and rs3918227 in the *NOS3* polymorphism present elevated levels of Cr and Cys-C, respectively. We concluded that *MYSM1* and polymorphisms in the *NOS3* gene might contribute to T2D risk in the Chinese Han population.

Although our study suffered from some limitations, such as a relatively small sample size and a lack of data on the lifestyle habits of patients (e.g., active smoking, quality of sleep, weight loss, and diet), our results indicated that seven SNPs in *MYSM1*, *PLXDC2*, *ARHGAP22*, and *HS6ST3*, increase T2D risk in the Chinese Han population. Functional studies on gene-gene and gene-environment interactions and clinical studies with larger sample sizes could further substantiate the impact of these gene polymorphisms on T2D.

## MATERIALS AND METHODS

### Patients and samples

Our sample consisted of 706 Chinese Han individuals: 319 patients with T2D and 387 controls with normal glucose tolerance (NGT). These participants were recruited between August 2010 and December 2014 from clinics at the department of Endocrinology, Xi’an NO.1 Hospital, Xi’an, China. The key inclusion criteria of case subjects by the World Health Organization in 2010 [43] were: (1) T2D diagnosis by a qualified respiratory physician; (2) impaired glucose tolerance (IGT) [2-h values in the oral glucose tolerance test (OGTT) exceed 199 mg/dl (11.0mmol/L); (3) hemoglobin A1C (HbA1C) test to diagnose diabetes, with a threshold of ≥ 6.5%, and (4) fasting plasma glucose levels of at least 126 mg/dl (7mmol/L in SI units). Individuals taking medication that affects glucose metabolism were excluded. Eligible controls were Chinese Han individuals residing in Northwest China. In addition, all patients gave informed consent and completed a personal interview. The participants provided information about anthropometric characteristics including height and weight to determine body mass index (BMI), as well as, age, sex, family history of diabetes and living habits. Midstream urine samples were collected to assess albumin and creatinine (Cr) levels. All participants consented to venipuncture for DNA and biochemical tests. The study was approved by the First Affiliated Hospital of Xi'an Jiaotong University.

### SNP selection and genotyping

We genotyped a total of 18 SNPs previously reported to be associated with T2D. *MYSM1* (rs2811893), *KIAA0825* (rs17376456), *PLXDC2* (rs1571942), rs12219125, and *ARHGAP22* (rs4838605) were selected from a GWAS concerning T2D in the Taiwanese population [17]. SNPs rs3007729, rs1342038, rs10927101, rs10199521, rs13064954, rs7772697, rs11765845, and rs10403021 were identified from a replication association study of diabetic retinopathy [18]. Finally, rs3025040, rs1799983, rs3918227, rs6219, and rs6214 were chosen from other T2D studies [19–21]. The above SNPs were identified by dbSNP (http://www.ncbi.nlm.nih.gov/projects/SNP). The MAF of all SNPs was > 5% in the HapMap of the Chinese Han Beijing (CHB) population. The SNPs were genotyped using Sequenom MassARRAY RS1000 (Sequenom, Inc.) containing: PCR amplification assay, designed primers and probes, purification by Shrimp Alkaline Phosphatase (SAP), followed by addition of primers and extension of the special basic group, followed by stimulation cocrystallization combining sample analyte and chip substrates. Primers were designed for each SNP using Primer3 (http://frodo.wi.mit.edu).

### Statistical analysis

All demographic parameters are presented as mean ± standard deviation (mean ± SD), unless otherwise specified. Comparisons of participants’ characteristics were performed using an independent sample *t*-test (using Levene's test for equality of variances) for continuous variables and an additional Chi-square test for categorical variables. All populations also underwent testing for Hardy-Weinberg equilibrium (HWE). We used the PLINK software package (version 1.07) to assess genetic data. A multivariate logistic regression model, adjusting for age and gender, was used to estimate risk associations between genotypes and T2D by computing the odds ratio (OR) and 95% confidence intervals (95% CIs). To investigate potential models of inheritance, the log-additive, over-dominant, dominant and recessive genotypic models were applied.
